# Molecular mechanisms underlying osteoarthritis development: Notch and NF-κB

**DOI:** 10.1186/s13075-017-1296-y

**Published:** 2017-05-15

**Authors:** Taku Saito, Sakae Tanaka

**Affiliations:** 1 Graduate School of Medicine, The University of Tokyo, 7-3-1 Hongo, Bunkyo-ku, Tokyo, 113-8655 Japan; 2 Bone and Cartilage Regenerative Medicine, Faculty of Medicine, The University of Tokyo, 7-3-1 Hongo, Bunkyo-ku, Tokyo, 113-8655 Japan

**Keywords:** Osteoarthritis, Articular chondrocyte, Notch, NF-κB

## Abstract

Osteoarthritis (OA) is a multi-factorial and highly prevalent joint disorder worldwide. Since the establishment of murine surgical knee OA models in 2005, many of the key molecules and signalling pathways responsible for OA development have been identified. Here we review the roles of two multi-functional signalling pathways in OA development: Notch and nuclear factor kappa-light-chain-enhancer of activated B cells. Previous studies have identified various aspects of articular chondrocyte regulation by these pathways. However, comprehensive understanding of the molecular networks regulating articular cartilage homeostasis and OA pathogenesis is needed.

## Background

Osteoarthritis (OA) is the most prevalent joint disease worldwide, causing chronic disability in older people. Various factors are associated with its pathogenesis, including aging, obesity, joint instability, and joint inflammation [1]. Since the establishment of experimental murine models with surgically induced knee joint instability [2, 3], many studies have revealed the major molecules or signalling pathways responsible for OA, such as a disintegrin-like and metallopeptidase with thrombospondin type 1 motif 5 (Adamts 5), matrix metalloproteinase-13 (Mmp13), hedgehog signalling, syndecan-4, Wnt signalling, and hypoxia-inducible factor 2-alpha (HIF-2α) [2, 4–10]. More recently, an increasing number of papers have reported involvement of molecules or signalling pathways that regulate various biological phenomena, including oxidative stress, autophagy, epigenetic change, cellular senescence, circadian rhythm, and microRNAs [11–17].

Adult articular chondrocytes have long been regarded as cycle-arrested cells that are unable to undergo proliferation. Hence, articular cartilage was previously referred to as “permanent cartilage” and was thought to lack the capability for self-restoration. Interestingly, a recent study using a Cre/loxP-based cell tracking technique has shown that proteoglycan-4 (Prg4)-expressing cells located in the superficial zone of adult articular cartilage expand to the middle zone above the tidemark, indicating that chondrocytes are slowly differentiating in adult articular cartilage [18]. Consequently, this novel finding is now innovating the concept of articular cartilage homeostasis.

Here, we focus on two multi-functional signalling pathways, Notch and nuclear factor kappa-light-chain-enhancer of activated B cells (NF-κB). Previous studies have reported various and somewhat contradictory effects of these two signalling pathways in chondrocytes. We primarily select studies in which roles of Notch or NF-κB were investigated using murine experimental OA models, and discuss the roles of these signalling pathways in OA pathophysiology and articular cartilage homeostasis, considering recent findings in articular cartilage.

## Notch signalling

Notch is a single-pass transmembrane cell surface receptor that plays a crucial role in cell-fate determination by regulating differentiation and apoptosis during embryogenesis and post-natal development [19, 20]. In mammals, the Notch signalling pathway consists of several molecules, including Notch ligands (Delta-like 1, 3 and 4, or Jagged (Jag) 1 and 2), Notch receptors (Notch 1–4), the transcriptional effector recombination signal binding protein for Ig kappa J (Rbpj), and target transcription factors hairy and enhancer of split (Hes) and hairy/enhancer-of-split related with YRPW motif (Hey) [20]. Notch signalling is initiated when Notch ligands on the cell surface bind to Notch receptors on adjacent cells. Upon ligand binding, the Notch receptor is cleaved by Adam and subsequently by a γ-secretase complex. Notch-intracellular domain (ICD) then translocates to the nucleus and binds to Rbpj to form a transcriptional activator that induces the Hes/Hey family.

We have reported previously that the activation of Notch signalling in articular chondrocytes contributes to the development of OA [21, 22]. Notch 1 and 2 receptors are highly expressed in articular chondrocytes [21] and are localized at the cell surface in normal mouse and human articular cartilage, but are translocated into the nucleus in degenerated cartilage [21]. Inhibition of Notch signalling by Rbpj knockout in chondrocytes after skeletal development suppresses OA development in a murine surgical model, and injection of the γ-secretase inhibitor DAPT into the knee joints of the wild-type OA model mice results in a similar protective effect [21]. Mmp13 expression is increased by overexpression of Notch-ICD in mouse primary chondrocytes and chondrocyte cell lines, and is decreased in Rbpj-knockout cartilage [21].

Among canonical Notch ligands, Jag1 is abundantly expressed and markedly increased during murine OA development [21]. Although nephroblastoma overexpressed (Nov, also known as Ccn3) and microfibril-associated glycoprotein 1 (MAGP1) are reported as non-canonical Notch ligands and are expressed in normal articular cartilage, their levels do not increase with the development of OA [21]. Increased expression of Jag1 may be involved in OA; however, its role as an OA trigger is not proven. Considering that articular chondrocytes are not in close contact with each other, ligand-independent activation of Notch signalling or ligand secretion may be associated with OA pathogenesis.

With respect to downstream signalling molecules, Hes1, Hes5, Hes7, Hey1, Hey2, and HeyL are well known to be direct transcriptional targets and mediate the effects of Notch signalling. However, only Hes1 is highly expressed in articular chondrocytes [21, 22]. Hes1 overexpression induces Mmp13 expression as well as Notch-ICD, and knockdown of Hes1 by siRNA cancels the increase of Mmp13 by Notch-ICD [21, 22]. This indicates that the catabolic effect of Notch signalling is mediated by Hes1. Furthermore, Hes1 knockout in adult mouse chondrocytes suppresses surgically induced OA with decreased expression of Mmp13 [22]. Although Hes1 itself is a known transcriptional repressor, it functions as a transcriptional activator in cooperation with calcium/calmodulin-dependent protein kinase II (CaMK2) [23]. In articular cartilage, CaMK2δ is abundantly expressed and required for transcriptional induction of catabolic genes by Hes1 [22]. Microarray analysis and chromatin immunoprecipitation and deep sequencing (ChIP-seq) have revealed that Hes1 induces many inflammation-related molecules such as interleukin (IL)-6 and IL-1 receptor-like 1 (IL1RL1, a receptor of IL-33), in addition to Mmp13 and Adamts5 [22]. Induction of these molecules by the Notch–Hes1 axis seems to be consistent with amelioration of murine collagen-induced arthritis by Notch inhibition, as reported recently by other researchers [24, 25].

In contrast to the mouse surgical OA model data, recent reports have shown that inhibition of Notch signalling results in accelerated OA progression with aging [26, 27]. In one study, Rbpj knockout in limb mesenchymal progenitor cells using Prx1-Cre, or in adult chondrocytes using Col2a1-Cre^ERT2^, promoted OA development within 8 months without surgical induction [26]. Similar results have been reported by the same group using Acan-Cre^ERT2^ and Rbpj-flox mice [27]. Interestingly, fibrotic cells and high levels of Mmp13 are observed in the superficial zone of Rbpj-knockout cartilage [27].

**Fig. 1:**
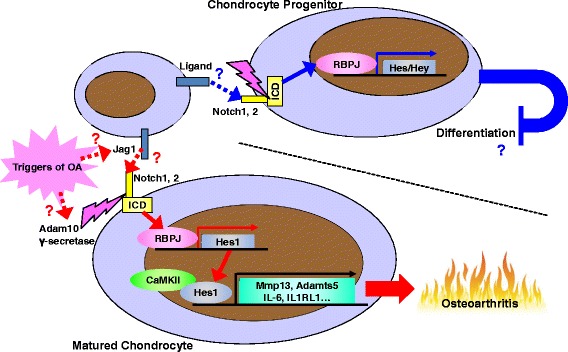
Hypothetical schematic of the physiological and pathophysiological roles of Notch signalling in articular cartilage. In mature chondrocytes, activation of the Notch–Rbpj–Hes1 pathway induces expression of catabolic and inflammation-related factors and enhances osteoarthritis (*OA*) development. Although Jag1 expression is abundant and increased with OA progression, the mechanisms underlying Notch activation in mature chondrocytes remain unclear. In contrast, Notch signalling may contribute to maintenance of chondrocyte progenitors and consequent homeostasis of articular cartilage. *Adamts5* A disintegrin and metallopeptidase with thrombospondin type 1 motif 5, *CaMKII* calcium/calmodulin-dependent protein kinase II, *Hes* hairy and enhancer of split, *Hey* hairy/enhancer-of-split related with YRPW motif, *ICD* intracellular domain, *IL* interleukin, *IL1RL1*IL-1 receptor-like 1, *Jag* Jagged, *Mmp* matrix metalloproteinase, *Rbpj* recombination signal binding protein for Ig kappa J

It is difficult to interpret the contradictory results of OA regulation by Notch signalling. Notch signalling regulates organogenesis by suppressing stem or progenitor cell differentiation [20]. In bone homeostasis, Notch signalling maintains a pool of mesenchymal progenitors in the bone marrow by suppressing osteoblast differentiation [28]. Meanwhile, Notch1 is highly expressed in chondrocytes of the superficial layer, and inhibition of Notch signalling abolishes the colony-forming ability of these cells [29]. Taken together, and including the recent paper highlighting articular chondrocyte turnover [18], we believe Notch signalling contributes to maintain progenitors whilst suppressing the regulation of chondrocyte progenitor differentiation. Furthermore, its mis-activation in differentiated chondrocytes in the middle and deep zones may lead to degeneration (Fig. 1).

OA development is not only caused by abnormalities of the articular cartilage, but also in the synovium, meniscus, ligament, and subchondral bone. Gamma-secretase inhibitor treatment suppresses production of proinflammatory cytokines from the synoviocytes of rheumatoid arthritis patients [25], implying that Notch signalling may contribute to enhanced production of inflammation-related molecules in OA synoviocytes as well as in chondrocytes. Because Notch signalling regulates bone homeostasis through maintenance of mesenchymal progenitors [28], it may be also associated with pathological changes of the subchondral bone, despite Notch signalling in mature osteoblasts not being involved in OA [27].

## NF-κB signalling

The NF-κB family has essential roles in a wide range of biological processes including cell survival, proliferation, differentiation, apoptosis, aging, inflammation, and immune responses [30–32]. Its members are v-rel reticuloendotheliosis viral oncogene homologue A (RelA, also known as p65), RelB, Rel, p105/p50, and p100/p52, each of which includes a Rel homology domain that mediates DNA binding and dimerization. These proteins form heterodimers and work as transcriptional activators. Inhibitors of NF-κB (IκB) proteins, such as IκBα, IκBβ, IκBγ, IκBε, IκBζ, and Bcl-3, bind to NF-κB family members in the cytoplasm [33]. Activation of IκB kinases (IKKs) in response to several signals phosphorylates IκB proteins and causes their degradation, which enables free NF-κB complexes to translocate from the cytoplasm into the nucleus and trigger target gene transactivation [34, 35]. NF-κB signalling is widely involved in OA pathophysiology through various effects and is activated in OA chondrocytes during aging and inflammation [36]. The NF-κB pathway is essential to induce various inflammation-related factors, including Mmp proteins, inducible nitric oxide synthase (iNOS), IL-1β, and TNF-α, and these induced cytokines further activate the signalling cascade [36].

HIF-2α is a NF-κB-related molecule that is essential for OA development and similar to HIF-1α is a transcription factor of the HIF family. HIF-1α protein is stabilized only under hypoxic conditions, and exerts various effects necessary for cell survival and adaptation in hypovascular and hypoxic tissues. In contrast, HIF-2α is expressed in well-vascularized tissues [37, 38]. HIF proteins share ~50% amino acid homology [39] and accumulating evidence indicates their distinct functions [39–43]. HIF-2α expression is increased in the middle and deep zones of articular cartilage [9] and surgically induced OA progression is markedly suppressed in HIF-2α hetero-knockout mice. Furthermore, various catabolic factors such as Mmp13, Mmp9, and vascular endothelial growth factor A (Vegfa) are down-regulated by HIF-2α haploinsufficiency, whilst others are directly induced by HIF-2α [9, 10]. HIF-2α is induced by pro-inflammatory cytokines IL-1β and TNF-α, and its induction is suppressed by treatment with IKK inhibitor [9, 44]. These data, combined with our promoter analyses, indicate that HIF-2α is a direct transcriptional target of NF-κB [9].

**Fig. 2:**
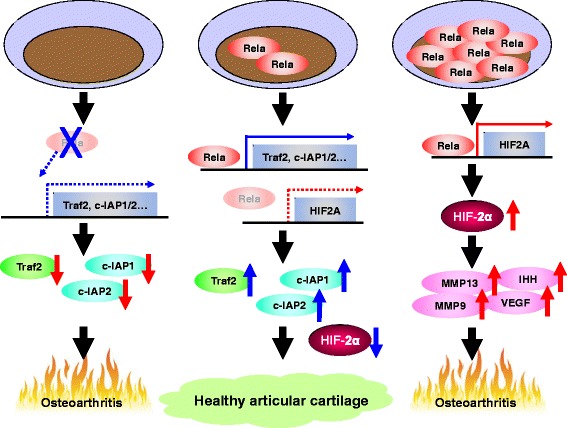
Regulation of articular cartilage by NF-κB signalling. Excessive activation of NF-κB signalling upregulates HIF-2α transcription, which results in enhanced OA progression through further induction of catabolic factors such as MMPs, VEGF, and IHH. In contrast, RelA knockout in chondrocytes causes deficiency of anti-apoptotic factors such as Traf2, c-IAP1, and c-IAP2, and leads to cartilage degeneration. Moderate activity of NF-κB signalling is necessary to maintain healthy articular cartilage. *HIF* hypoxia-inducible factor, *IHH* indian hedgehog, *Mmp* matrix metalloproteinase, *Rela* v-rel reticuloendotheliosis viral oncogene homologue A, *VEGF* vascular endothelial growth factor

In addition to its catabolic effects in chondrocytes, NF-κB signalling plays an essential role in cell survival. We next examined the physiological and pathophysiological roles of NF-κB in articular cartilage. RelA is predominantly localized in the cytoplasm of normal articular chondrocytes and is translocated into the nucleus of OA chondrocytes, accompanied with phosphorylated IκBα [44]. In-vivo analyses using Col2a1-Cre^ERT^ and Rela-flox mice have revealed that RelA homozygous knockout in chondrocytes after skeletal growth leads to marked acceleration of OA, whereas RelA heterozygous knockout suppresses OA progression [44]. Apoptotic cells are increased in homozygous-knockout cartilage, and anti-apoptotic genes such as Traf2, c-IAP1, and c-IAP2 are down-regulated by RelA deficiency [44]. Notably, HIF-2α expression is suppressed in both homozygous and heterozygous knockout cartilage, but the anti-apoptotic genes are not down-regulated in heterozygous knockout cartilage. Although the anti-apoptotic genes and HIF-2α are transcriptional targets of RelA, the former factors are induced by a smaller amount of RelA than the latter [44]. Thus, NF-κB signalling in chondrocytes is critical for cartilage homeostasis and OA development (Fig. 2).

Numerous studies have revealed that NF-κB signalling is involved in inflammatory responses. Various pro-inflammatory factors secreted from articular chondrocytes and synovial cells modulate OA development, and NF-κB signalling plays essential roles in mediating their effects [45]. In addition, NF-κB signalling regulates some responses to mechanical loading [46]. Dynamic compressive strain enhances phosphorylation of IκBα and subsequent intra-nuclear translocation of RelA in a magnitude-dependent manner without IL-1β treatment, whereas low magnitude strain suppresses this with IL-1β treatment [47]. Considering that excessive mechanical loading is the major risk factor for OA [1], elucidation of the molecular mechanisms underlying the mechano-responses regulated by NF-κB signalling may lead to a more comprehensive understanding of OA pathophysiology. Furthermore, the NF-κB–HIF-2α axis may be a potent therapeutic target because haploinsufficient RelA or a low dose of an IKK inhibitor can suppress HIF-2α expression without significant effect on cell survival [44].

## Prospects

**Fig. 3:**
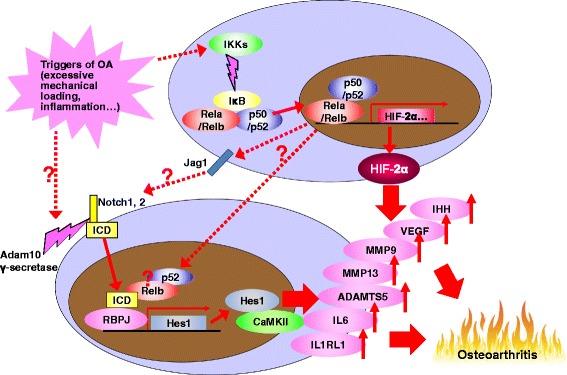
Crosstalk between Notch and NF-κB signalling pathways in regulation of OA development. Increase of Jag1 expression during OA progression may be due to activation of NF-κB signalling, as well as occurring in endothelial cells. The non-canonical NF-κB proteins p52 and RELB may enhance transcriptional activity of Notch-ICD and Rbpj, as seen in rheumatoid arthritis model mice. Among the identified downstream factors, proteinases and inflammation-related molecules are probably common mediators of both pathways. *Adamts5* A disintegrin and metallopeptidase with thrombospondin type 1 motif 5, *CaMKII* calcium/calmodulin-dependent protein kinase II, *Hes* hairy and enhancer of split, *HIF* hypoxia-inducible factor, *ICD* intracellular domain, *IHH* indian hedgehog, *IκB* inhibitors of NF-κB, *IKK* inhibitors of NF-κB kinase, *IL* interleukin, *IL1RL1*IL-1 receptor-like 1, *Jag* Jagged, *MMP* matrix metalloproteinase, *OA* osteoarthritis, *Rbpj* recombination signal binding protein for Ig kappa J, *Rela* v-rel reticuloendotheliosis viral oncogene homologue A, *VEGF* vascular endothelial growth factor

Many cells and tissues are regulated by complex multi-functional signalling pathways. In addition to Notch and NF-κB, regulation of articular cartilage by the canonical Wnt signalling pathway remains contradictory. In articular chondrocytes, OA development is enhanced by activation of canonical Wnt signalling in Col2a1-Cre^ERT2^;β-catenin^fx(ex3)/wt^ mice [8]; however, it is also enhanced by inhibition of Wnt signalling using Col2a1-ICAT transgenic mice, in which inhibitor of β-catenin and T cell factor (ICAT) is overexpressed under the Col2a1 promoter [4]. In contrast to these data in adult mice, activation of canonical Wnt signalling in chondrocytes enhances superficial zone thickness during the skeletal growth period, while conditional ablation of β-catenin causes the opposite result [48]. These data imply that canonical Wnt signalling exerts various age-dependent and locus-dependent effects. However, the in-depth processes of chondrocyte regulation by the Notch, NF-κB, and Wnt pathways remain ambiguous, and appear to be context dependent. Although these pathways are known to be involved in transcriptional induction of *Mmp13* and *Adamts5*, understanding how they regulate each other is important. Because transcription of Jag1 is enhanced by NF-κB in endothelial cells [49], activation of NF-κB signalling may precede that of Notch signalling. In rheumatoid arthritis model mice, Notch is activated by TNF and inhibits osteoblast differentiation in cooperation with the non-canonical NF-κB proteins p52 and RELB [50]. These findings imply that mutual interactions occur between these pathways during OA development, although the exact mechanisms remain unidentified (Fig. 3). Determination of the signalling networks that regulate maintenance of the progenitors, their differentiation to mature chondrocytes, and turnover of articular chondrocytes will be necessary to better understand OA pathophysiology.

In addition to these findings obtained from molecular biology experiments using murine models, we should address another essential issue: how these signalling pathways regulate human articular cartilage and OA. Many experimental studies using human samples have shown that activation of NF-κB signalling in chondrocytes and synovial cells is closely involved in enhanced production of inflammatory cytokines or catabolic enzymes [51]. Similarly, previous studies have shown enhanced expression of Notch-related molecules in human OA cartilage [52]. These data imply involvement of these signalling pathways in the pathophysiology of both human and murine OA. To date, however, NF-κB-related or Notch-related molecules have not been identified as OA susceptibility genes by previous genome-wide association analyses.

## Conclusion

The Notch and NF-κB signalling pathways regulate articular chondrocyte homeostasis and OA development in various ways. Comprehensive understanding of the molecular networks including articular chondrocyte differentiation is needed for further elucidation of OA pathogenesis.

## Abbreviations

Adam: A disintegrin and metalloproteinase
Adamts5: A disintegrin and metallopeptidase with thrombospondin type 1 motif ﻿5﻿
CaMK2: Calcium/calmodulin-dependent protein kinase II
ChIP-seq: Chromatin immunoprecipitation and deep sequencing
Hes: Hairy and enhancer of split
Hey: Hairy/enhancer-of-split related with YRPW motif
HIF-2α: Hypoxia-inducible factor 2 alpha
ICAT: Inhibitor of β-catenin and T cell factor
ICD: Intracellular domain
IKK: IκB kinase
iNOS: Inducible nitric oxide synthase
IκB: Inhibitors of NF-κB
MAGP1: Microfibril-associated glycoprotein 1
Mmp13: Matrix metalloproteinase 13
NF-κB: Nuclear factor kappa-light-chain-enhancer of activated B cells
Nov: Nephroblastoma overexpressed gene
OA: Osteoarthritis
Prg4: Proteoglycan-4
Rela: V-rel reticuloendotheliosis viral oncogene homologue A
TGF-β: Transforming growth factor beta
Vegfa: Vascular endothelial growth factor A
